# Analysis of copy number alterations in bladder cancer stem cells revealed a prognostic role of *LRP1B*

**DOI:** 10.1007/s00345-022-04093-1

**Published:** 2022-07-16

**Authors:** Donatella Conconi, Andrea Jemma, Martina Giambra, Serena Redaelli, Giorgio Alberto Croci, Leda Dalprà, Marialuisa Lavitrano, Angela Bentivegna

**Affiliations:** 1 School of Medicine and Surgery, University of Milano-Bicocca, Monza, 20900 Italy; 2 PhD Program in Neuroscience, University of Milano-Bicocca, Monza, 20900 Italy; 3 Pathology Unit, Fondazione IRCCS Ca’ Granda Ospedale Maggiore Policlinico, Milan, 20122 Italy

**Keywords:** Bladder cancer, Copy-number alterations, Cancer stem cells (CSCs), Predictive CNAs, Prognosis, LRP1B

## Abstract

**Purpose:**

Bladder cancer is the most common malignancy of the urinary tract and one of the most prevalent cancers worldwide. It represents a spectrum of diseases, from recurrent non-invasive tumors (NMIBCs) managed chronically, to muscle infiltrating and advanced-stage disease (MIBC) that requires multimodal and invasive treatment. Multiple studies have underlined the complexity of bladder tumors genome, highlighting many specific genetic lesions and genome-wide occurrences of copy-number alterations (CNAs). In this study, we analyzed CNAs of selected genes in our cohorts of cancer stem cells (CSCs) and in The Cancer Genome Atlas (TCGA-BLCA) cohort with the aim to correlate their frequency with patients’ prognosis.

**Methods:**

CNAs have been verified on our array-CGH data previously reported on 19 bladder cancer biopsies (10 NMIBCs and 9 MIBCs) and 16 matched isolated CSC cultures. In addition, CNAs data have been consulted on the TCGA database, to search correlations with patients’ follow-up. Finally, mRNA expression levels of *LRP1B* in TGCA cohort were obtained from The Human Protein Atlas.

**Results:**

We firstly identified CNAs differentially represented between TGCA data and CSCs derived from NMIBCs and MIBCs, and we correlated the presence of these CNAs with patients’ follow-up. *LRP1B* loss was significantly increased in CSCs and linked to short-term poor prognosis, both at genomic and transcriptomic level, confirming its pivotal role in bladder cancer tumorigenesis.

**Conclusion:**

Our study allowed us to identify potential "predictive" prognostic CNAs for bladder cancer, implementing knowledge for the ultimate goal of personalized medicine.

**Supplementary Information:**

The online version contains supplementary material available at 10.1007/s00345-022-04093-1.

## Introduction

Bladder cancer (urothelial cancer of the bladder) is the 10th most widespread cancer worldwide, with an estimated 549,000 new cases and 200,000 deaths reported in 2018 [1]. It can be classified according to its invasiveness as either non-muscle-invasive bladder cancer (NMIBC) or muscle-infiltrating bladder cancer (MIBC) [1].

Approximately 75% of newly diagnosed patients have papillary NMIBCs [2]. NMIBCs frequently recur (50–70%) but infrequently progress to invasion (10 –15%), and 5-year survival is nearly 90%. In contrast, about 20% of tumors show muscle infiltration at diagnosis, with 5-year survival less than 50% [3]. Although multiple studies tried to differentiate NMIBC and MIBC also at the molecular level, a few advances in clinical management have been made over the past decades [4].

Array-based comparative genomic hybridization technologies have been necessary in highlighting genome-wide occurrences of copy-number alterations (CNAs). Over 20% of non-invasive bladder tumors have been indicated to have CNAs compared to 30% of infiltrating bladder tumors [5]. Therefore, revealing specific chromosomal regions related to patients’ survival or to the potential tumor progression may allow the introduction of molecular CNAs profiling as part of clinical strategy for progression risk evaluation, thus implementing the personalized therapeutic approach [5]. In this context, the study of cancer stem cells as the main responsible for tumor initiation and recurrences could be crucial.

**Fig. 1:**
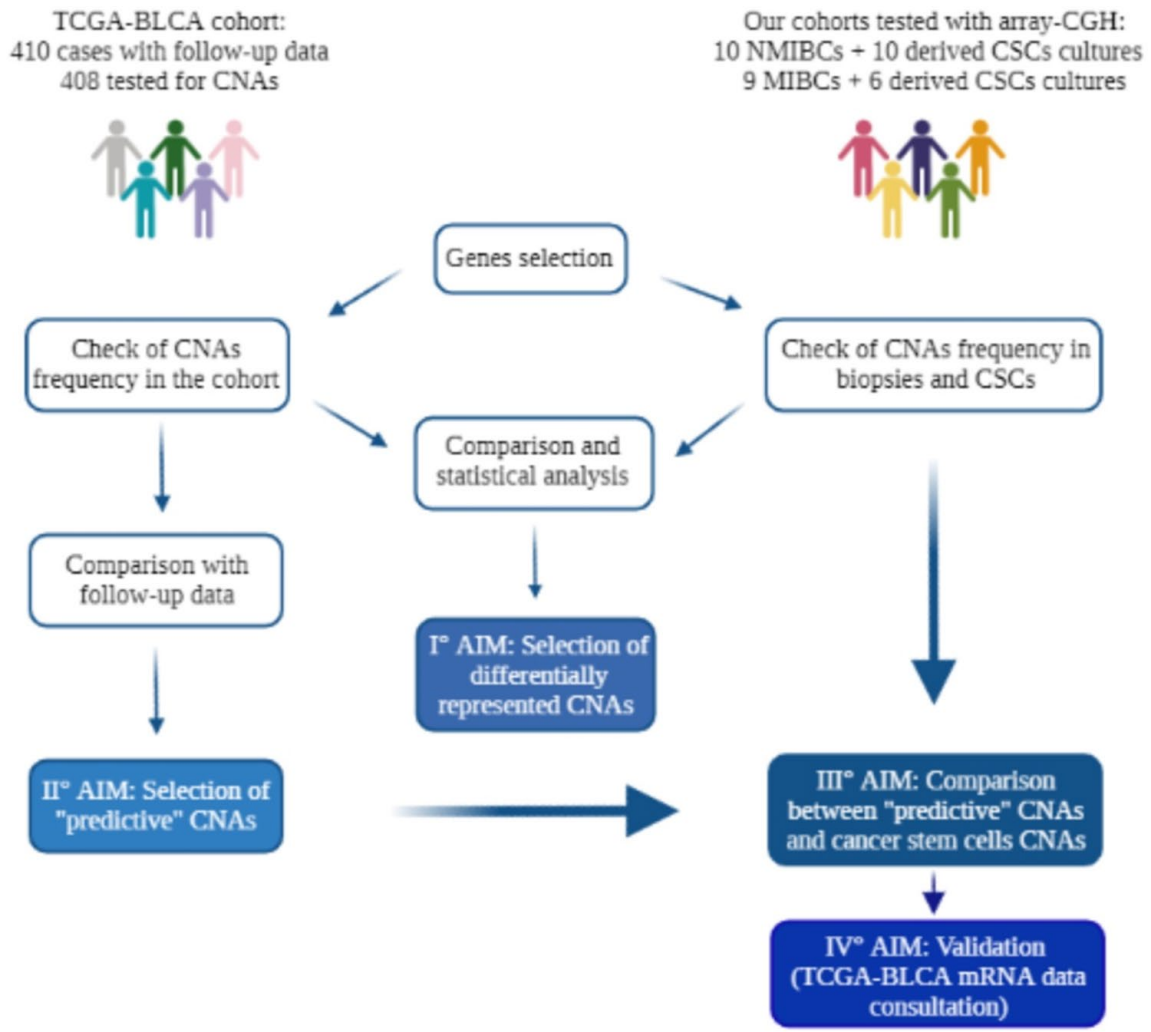
Created with BioRender.com (accessed on 22 March 2022)

In this study, we analyzed the CNAs occurrences of selected genes in our cohorts of tumor samples, in derived cancer stem cells and in The Cancer Genome Atlas (TCGA-BLCA) cohort, with the aim to correlate their frequency with patients’ prognosis, to identify potential “predictive” prognostic CNAs for bladder cancer (Fig. 1).

## Materials and methods

### Tumor samples, CSCs and array comparative genomic hybridization data

Bladder cancer specimens were collected from 19 patients that underwent transurethral resection, as previously reported [6, 7]. Staging and grading were done according to the AJCC 8th edition by a pathologist (see Table S1a for all details).

Cancer stem cells (CSCs) were previously isolated and characterized [7, 8]. Array Comparative Genomic Hybridization (Array-CGH) experiments were described in [6, 7]. More details are in Supplemental Information.

### TCGA GDC data portal analysis

CNAs’ data from human bladder cancer samples were obtained from the TCGA database https://portal.gdc.cancer.gov/ (last access date: 22 December 2021). In particular, we selected the TCGA-BLCA project, composed of 410 samples (see Table S1b for histological characteristics). Patients’ vital status and history of prior cancer diagnosis were reported. Data related to progression-free survival were not available. CNAs data were available for 408 cases out of 410. Frequency of CNAs was calculated as the number of samples with CNAs for a gene/number of total tested samples (*n* = 408). Alive and dead patients’ number was obtained by the Vital Status field. Time-point survival was achieved by setting different “Days to the Dead” field.

### The human protein atlas

mRNA expression levels of *LRP1B* in TGCA cohort were obtained from The Human Protein Atlas (https://www.proteinatlas.org/ENSG00000168702-LRP1B/pathology/urothelial+cancer, last access date: 9 March 2022). RNA-seq data are reported as average FPKM (fragments per kilobase of exon per million mapped reads). Based on the FPKM values, patients were classified into two groups "low" (under cut off) or "high" (over cut off) expression. Selected cut-off value was “best expression cut-off” (0.01) indicated for LRP1B.

## Results

### First aim: identification of CNAs differentially represented between TCGA GDC data and CSCs

We first identified 34 genes involved in copy-number aberrations in bladder cancer. The selection was based on literature (see Supplemental Information) and our previous studies [6, 7]. Copy number of these genes was checked on our array-CGH data of 19 bladder cancer biopsies (10 NMIBCs and 9 MIBCs) and 16 matched isolated cancer stem cells cultures (Table S2, [7]). The frequency of CNAs in these genes is reported in Table S3.

**Fig. 2:**
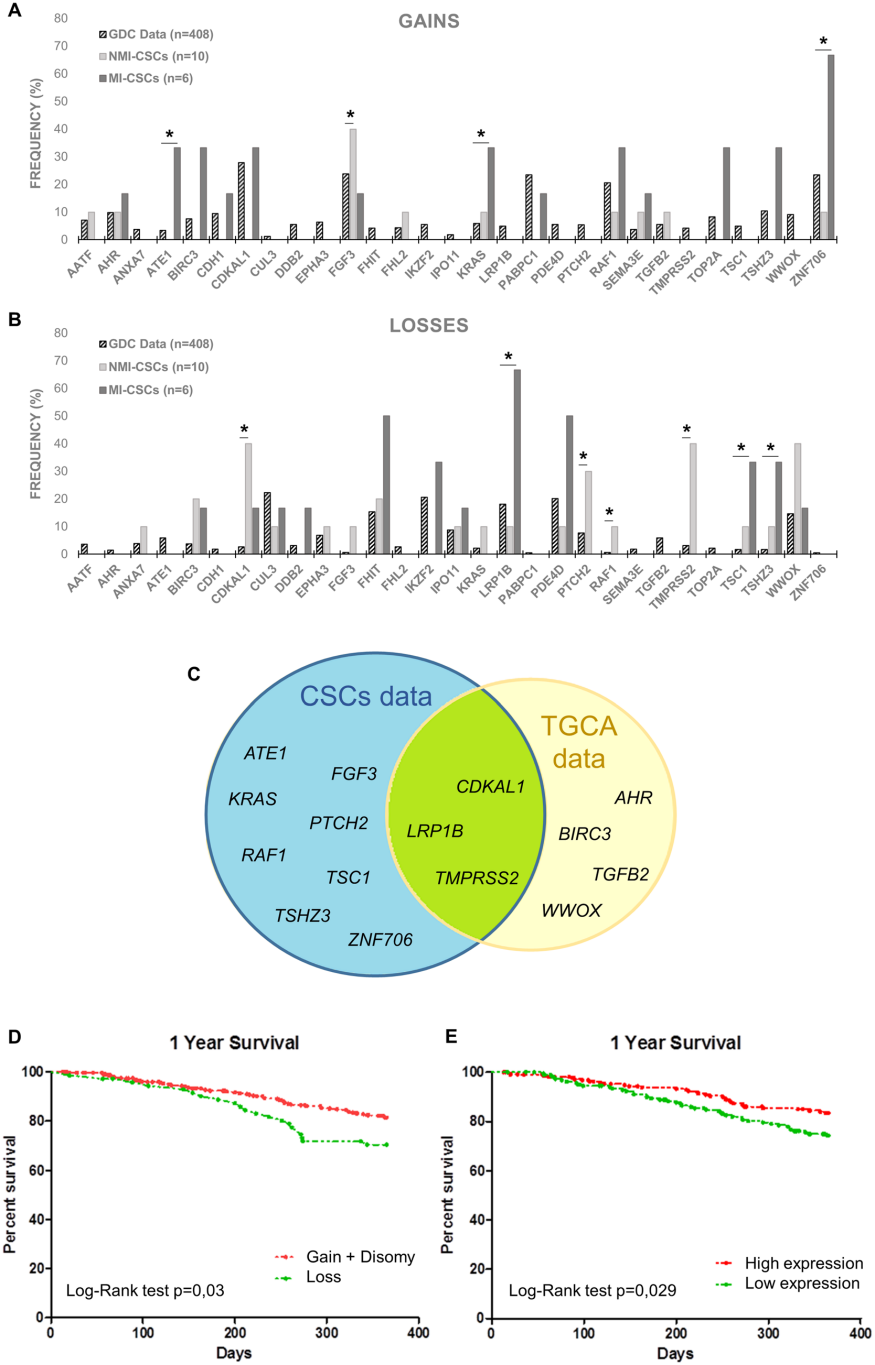
**A**, **B** CNAs frequency in TCGA GDC Data Portal and CSCs cultures. *NMI* non-muscle-invasive, *MI* muscle-infiltrating, *CSCs* cancer stem cells. *p < 0.05 Chi-square or Fisher's exact test. **C** Schematic representation of CNAs data in the two cohorts. **D** Kaplan–Meier plots showing 1-year survival of patients with loss or disomy/gain of LRP1B. **E** Kaplan–Meier plots showing 1-year survival of patients with low or high *LRP1B* RNA expression

Then, we compared CNAs frequencies with those reported in TCGA GDC Data Portal referred to TCGA-BLCA project (number of cases where CN gain or loss was observed/408 cases tested for CNAs in every gene, https://portal.gdc.cancer.gov). Since the BLCA project is mainly composed of MIBCs (98,7% of samples) and NMIBC data are almost absent, we compared both cancer histotypes with the MIBCs’ dataset. Statistical analysis between database data and our results revealed significant differences in frequency of some CNAs (Table S3, Chi-square or Fisher's exact test). Due to CSCs pivotal role in cancer progression and recurrence, we focused our attention on CNAs differentially represented between GDC data and our isolated CSCs, identifying genes with a CN gain or loss preferentially in this subpopulation (*ATE1*, *FGF3*, *KRAS*, *ZNF706* and *CDKAL1*, *LRP1B*, *PTCH2*, *RAF1*, *TMPRSS2*, *TSC1*, *TSHZ3* respectively, Fig. 2A, B).

### Second aim: correlation of CNAs with patients’ follow-up

To overcome the absence of follow-up data from our patients and to increase the number of cases, we consulted The Cancer Genome Atlas (BLCA project). We extracted survival data from the TCGA-BLCA population (410 samples) after setting three time-points for survival (6 months, 1 year and 5 year). We reported the survival data of patients carrying copy-number gain/loss in genes reported in Table S4. We expressed this information as the ratio of the number of alive and/or dead patients to the number of cases carrying copy-number gain and/or loss in that gene. Then, we compared the survival data of patients carrying a specific CNA with those of the total cohort (Table S4).

Statistical analysis (Chi-square or Fisher's exact test) revealed significant differences in survival in several CNAs carriers, suggesting a possible role of these genes in patients’ prognosis. In particular, the percentage of dead patients in *WWOX* loss carriers was significantly higher than the percentage of dead patients in the total cohort at 6 months (Chi-square test, *p* < 0.05). Similarly, the loss of *LRP1B* seemed to correlate with a worse 1-year survival (Chi-square test, *p* < 0.05). Conversely, gain of *BIRC3* and *CDKAL1* genes and loss of *AHR* were associated with a better 5-year survival (Chi-square or Fisher's exact test, *p* < 0.05).

The same approach was used to check the presence of CNAs associated with tumor relapse. Unfortunately, progression-free survival data are not available in the database, so we analyzed the presence of prior malignancy in the complete cohort versus CNAs’ carrier groups. Even in this case, we identified CNAs in genes potentially related to tumor recurrence, such as *TGFB2* and *TMPRSS2*.

### Third aim: comparison of “predictive” CNAs and CSCs data

Comparison of “predictive” CNAs and CSCs data revealed that *LRP1B* loss was also significantly more represented in CSCs, confirming its prominent role in bladder cancer (Fig. 2B, C). Moreover, *CDKAL1* loss was significantly increased in NMIBCs-derived CSCs, indirectly supporting TGCA data previously shown (Fig. 2B, C). *TMPRSS2* loss was also significantly increased in NMI-CSCs; however, TGCA data showed an association of both CN gain and loss with tumor recurrence (Table S4).

### Fourth aim: RNA expression data consultation

Finally, mRNA expression levels of *LRP1B* in TGCA cohort were obtained from The Human Protein Atlas. Based on the expression level (fragments per kilobase of exon per million mapped reads—FPKM), patients were classified into two groups "low" or "high" expression (see Materials and methods). Analysis of correlation between mRNA expression levels and 1-year survival confirmed the prognostic role of LRP1B not only at the genomic but also at the transcriptional level (Fig. 2D, E). Tumor stage classification did not highlight a subgroup with a better correlation between *LRP1B* expression and patient prognosis (data not shown).

## Discussion

Despite several improvements in bladder cancer knowledge, it is still the tenth most common cause of cancer death and about 25% of these tumors are muscle infiltrating or metastatic at diagnosis, mostly with a poor prognosis [9].

The large progress in technology development has allowed a deeper understanding of the molecular mechanisms regulating bladder cancer development and progression. Furthermore, recursive use of genomics and transcriptomics in clinical settings determines the prospect of precision medicine, which requires the identification of accurate elements for assessing patients’ response to therapy, prognosis and the recurrence frequency [10]. According to this, in the current work, we aimed to identify genes involved in copy-number alterations potentially related to patients’ prognosis.

First, analysis of CNAs frequencies in the TGCA cohort and our samples (primary tumors and isolated CSCs) allowed the identification of genes affected by differentially represented CNAs. In particular, *ATE1*, *FGF3*, *KRAS*, *ZNF706* showed a higher percentage of CN gain in the CSCs than in TGCA data.

Among these, the *ZNF706* gene, located at 8q22.3, is a zinc finger gene family member, involved in the transcriptional regulation of gene expression [11]. It was found to be up-regulated in laryngeal squamous cancer tissues and gastric cancers with amplification at 8q22.3 [11]. It also appeared up-regulated in breast tumors and it was indicated as a candidate biomarker of breast cancer progression [12]. Accordingly, we identified a strong increase of CN gain percentage in CSCs derived from MIBCs. Similarly, gain of *KRAS* was significantly enhanced in MIBCs-derived CSCs. We also found an increased percentage of CN gain of *FGF3* in NMIBCs-derived CSCs. *FGF3* has been repeatedly found amplified in bladder cancer and its up-regulated expression has been associated with tumor's malignant clinical phenotypes [13].

Concerning genes involved in CN losses preferentially found in CSCs, some are exclusive of NMIBCs, such as *CDKAL1*, *PTCH2*, *RAF1*, *TMPRSS2*, others of MIBCs (*LRP1B*, *TSC1*, *TSHZ3*). Some of these losses were identified as “predictive” by correlating their presence with patients’ follow-up using the TCGA GDC Data Portal.

Data evaluation uncovered some CNAs associated with tumor recurrence, in particular, gain of *TGFB2* and gain or loss of *TMPRSS2* (Table S4). Supporting this observation, TGFB2–Smad3 pathway has been demonstrated to promote proliferation and epithelial–mesenchymal transition in several cancers, recently also in bladder cancer [14, 15]. TMPRSS2, rather, has been reported for its contribution to severe acute respiratory syndrome coronavirus 2 (SARS-CoV-2) infection, but recently its role in different types of cancers was investigated [15]. The expression of *TMPRSS2* was found significantly decreased in many tumors, and correlated with poor prognosis [16]. Our findings demonstrated a significant increase of CN loss percentage in NMI-CSCs; however, an association of both gain and loss with tumor recurrence was discovered in TGCA data.

Data examination also allowed the identification of CNAs associated with patients’ survival. CN gain of *BIRC3* and *CDKAL1* genes and loss of *AHR* were associated with better 5-year survival. Amplifications of aryl hydrocarbon receptor (AHR) have been recently identified in bladder cancer [17], and its activation was also associated with grade, stage, and progression [18], confirming the protective role of its loss (100% of patients) observed in TGCA. We did not find a different frequency in CSCs. Similarly, no relevant differences in percentage of gain or loss were identified in CSCs for *BIRC3* gene.

*CDKAL1* gene maps at 6p22 where a highly prevalent amplification in bladder cancer compared to other cancer types has been registered [19]. However, knockdown of CDKAL1 did not affect cell proliferation [18], suggesting that it could exert its oncogenic properties in other ways, maybe by a co-amplification of the neighbor *E2F3* [20]. Interestingly, TGCA analysis uncovered a correlation between *CDKAL1* gain and better survival, and a significant increment of CN loss was observed in NMIBCs-derived CSCs, indirectly supporting TGCA results.

The second time-point analysis evidenced a correlation between CN loss of low-density lipoprotein receptor-related protein 1B (*LRP1B*) and a worst 1-year survival. *LRP1B*, member of the LDL receptor family, is a large gene located on chromosome 2q, containing more than 91 exons and spanning over 500 kb. The related protein is 4599 amino acids long [21] and it is involved in a large functional spectrum, ranging from receptor-mediated endocytosis, lipoprotein trafficking, transportation of nutrients and vitamins, developmental processes to cellular signaling [22]. *LRP1B* is one of the most altered genes in human cancer overall [22] and its role has been closely related to cancer progression. *LRP1B* gene was identified as commonly inactivated in non-small-cell lung cancer cell lines and its inactivation by several genetic and epigenetic mechanisms has been frequently reported in multiple tumor types [22], corroborating its proposed tumor suppressor function. Recently, *LRP1B* association with tumor mutational burden and immunotherapy efficacy has been confirmed in lung and hepatocellular carcinomas, revealing the gene mutational status as a potential biomarker for immune check-point inhibitors (ICI) treatment response, through mast cells activation and infiltration in tumor tissues [23, 24].

*LRP1B* role as a candidate tumor suppressor also for bladder cancer has been proposed several years ago [25]; however, its function still remains to be fully elucidated. A lower frequency of its copy-number deletions was newly associated with a specific subtype of bladder cancer (quiescent type) that exhibited the best overall survival [26]. Our TCGA analysis confirmed its crucial role, for the first time linked to short-term prognosis (1-year survival). An additional confirmation was given by the presence of a CN loss in 66.7% of MI-derived CSCs, significantly increased respect to TGCA data and biopsies. *LRP1B* prognostic role, in terms of expression level, through RNA-seq data analysis was also confirmed.

Finally, 6-month survival data examination revealed *WWOX* loss as a negative prognostic factor. WWOX is known as a global modulator of gene expression and cell metabolism [27]. It is one of the largest human genes and contains the second most common chromosomal fragile site FRA16D, a hot spot of genomic instability. This makes *WWOX* prone to breakage and frequent target for CNAs in cancer [28]. In fact, decrease or loss of its expression was found in several tumor tissues, and it is often correlated with higher tumor grade and unfavorable outcome [29]. Studies on bladder cancers revealed a critical role of *WWOX* in the tumorigenesis and loss of its protein expression correlates with higher tumor grade, more advanced stage, and shorter progression-free survival or overall survival [27–30]. According to these data, we found a high percentage of *WWOX* CN loss in MI-biopsies, not maintained in CSCs subpopulation.

## Conclusions

In conclusion, we first identified CNAs differentially represented between GDC data and CSCs derived from NMIBCs and MIBCs, and we correlated the presence of these CNAs with patients’ follow-up. *LRP1B* loss was significantly increased in CSCs and for the first time linked to short-term prognosis (1-year survival), both at genomic and transcriptomic level, confirming its pivotal role in bladder cancer tumorigenesis. Moreover, its recently identified role in the ICI response could have relevant clinical implications for the patients’ treatment in the future. However, the limitations of this study, such as the absence of follow-up data from our patients’ cohort and the limited number of CSC samples, indicate the need for further studies.

## Supplementary Information

Below is the link to the electronic supplementary material.

Supplementary file1 (DOCX 21 KB)

Supplementary file2 (DOCX 22 KB)

Supplementary file3 (XLSX 91 KB)

Supplementary file4 (DOCX 30 KB)

Supplementary file5 (DOCX 31 KB)
