# Supplementary material for: Analysis of copy number alterations in bladder cancer stem cells revealed a prognostic role of LRP1B
Source: World J Urol. 2022 Jul 16;40(9):2267–73. doi: 10.1007/s00345-022-04093-1 (PMC9287687; doi:10.1007/s00345-022-04093-1)
Supplement: Supplementary file 1 — Supplementary file1 (DOCX 21 KB) [file 345_2022_4093_MOESM1_ESM.docx]

Analysis of copy number alterations in bladder cancer stem cells revealed a prognostic role of *LRP1B*

Donatella Conconi, Andrea Jemma, Martina Giambra, Serena Redaelli, Giorgio Alberto Croci, Leda Dalprà, Marialuisa Lavitrano, Angela Bentivegna

**Supplemental Information.**

**Methods: Array Comparative Genomic Hybridization (Array-CGH)**

Genomic DNA extraction, sample preparation and slide hybridization were performed using SurePrint G3 Human CGH Microarray 8x60K (Agilent Technologies, Santa Clara, CA, USA) following the manufacturer’s recommendations. The arrays were scanned at 2-µm resolution and analysed using Feature Extraction v10.7 and Agilent Genomic Workbench v5.0 software (Agilent Technologies, Santa Clara, CA, USA). Aberration Detection Method 2 (ADM-2) algorithm was used to compute and assist the identification of aberrations in a given sample (threshold = 5.0), assigning a statistical score based on the average quality weighted log ratio (DLRS) of the sample and reference channels. We applied a filtering option of a minimum of three aberrant consecutive probes and a minimum absolute average log2 ratio that differs among all samples and depends on DLRS values, so it is related to the quality of the experiment. Log2ratio values over 1, which correspond to four copies, identify amplification; values under −1.7, which correspond to 0.6 copy, identify complete loss. The estimated percentage of mosaicism was assessed by the formula determined by Cheung et al [1]. In particular, log2ratio values over 0.6, which correspond to three copies, identify non-mosaic gains; values under −1, which correspond to 1 copy, identify non-mosaic losses. Accordingly, log2 ratio values for mosaic gains range between the DLRS value and 0.6 and for mosaic losses between the DLRS value and −1.

1. Cheung SW, Shaw CA, Scott DA, et al (2006) Microarray-Based CGH Detects Chromosomal Mosaicism Not Revealed by Conventional Cytogenetics. Am J Med Genet 221:212–221. https://doi.org/10.1002/ajmg.a

**Methods: selection of 34 genes involved in copy number aberrations (CNAs) in bladder cancer**

The selection was based on literature search using the keywords “bladder cancer”, “copy number alterations”, “CNAs”, “array-CGH”, “genomic alterations”.

Selected references:

- Lindquist KJ, Sanford T, Friedlander TW, et al (2017) Copy number gains at chr3p25 and chr11p11 are associated with lymph node involvement and survival in muscle-invasive bladder tumors. PLoS One 12:1–14. https://doi.org/10.1371/journal.pone.0187975
- Soave A, Chun FKH, Hillebrand T, et al (2017) Copy number variations of circulating, cell-free DNA in urothelial carcinoma of the bladder patients treated with radical cystectomy: A prospective study. Oncotarget 8:56398–56407. https://doi.org/10.18632/oncotarget.17657
- Zhao J, Xu W, He M, et al (2016) Whole-exome sequencing of muscle-invasive bladder cancer identifies recurrent copy number variation in IPO11 and prognostic significance of importin-11 overexpression on poor survival. Oncotarget 7:75648–75658. https://doi.org/10.18632/oncotarget.12315
- Bellmunt J, Kim J, Reardon B, et al (2021) Genomic predictors of good outcome, recurrence, or progression in high-grade T1 non-muscle-invasive bladder cancer. Cancer Res 80:4476–4486. https://doi.org/10.1158/0008-5472.CAN-20-0977
- Nascimento e Pontes MG, da Silveira SM, de Souza Trindade Filho JC, et al (2013) Chromosomal imbalances in successive moments of human bladder urothelial carcinoma. Urol Oncol Semin Orig Investig 31:827–835. https://doi.org/10.1016/j.urolonc.2011.05.015
- Weinstein JN, Akbani R, Broom BM, et al (2014) Comprehensive molecular characterization of urothelial bladder carcinoma. Nature 507:315–322. https://doi.org/10.1038/nature12965
- Robertson AG, Kim J, Al-Ahmadie H, et al (2017) Comprehensive Molecular Characterization of Muscle-Invasive Bladder Cancer. Cell 171:540-556.e25. https://doi.org/10.1016/j.cell.2017.09.007
- Hurst CD, Platt FM, Taylor CF, Knowles MA (2012) Novel tumor subgroups of urothelial carcinoma of the bladder defined by integrated genomic analysis. Clin Cancer Res 18:5865–5877. https://doi.org/10.1158/1078-0432.CCR-12-1807
