# Supplementary material for: Analysis of copy number alterations in bladder cancer stem cells revealed a prognostic role of LRP1B
Source: World J Urol. 2022 Jul 16;40(9):2267–73. doi: 10.1007/s00345-022-04093-1 (PMC9287687; doi:10.1007/s00345-022-04093-1)
Supplement: Supplementary file 2 — Supplementary file2 (DOCX 22 KB) [file 345_2022_4093_MOESM2_ESM.docx]

**Table S1a**. Patients’ data.

| PZ | SEX | AGE AT SURGERY | HISTOTYPE | GRADE | AJCC PATHOLOGIC T | INVASION | CLINICAL DATA | SAMPLE | |
| --- | --- | --- | --- | --- | --- | --- | --- | --- | --- |
|  |  |  |  |  |  |  |  | **B** | **CSC** |
| #1 | M | 81 | Non-invasive papillary urothelial carcinoma | low | pTa | no | Recurrence of a previuos NMI carcinoma. No follow-up and vital status information. | + | + |
| #2 | M | 79 | Non-invasive papillary urothelial carcinoma | high | pTa | no | Several subsequent NMI relapses.  No vital status information. | + | + |
| #3 | F | 56 | Non-invasive papillary urothelial carcinoma | low | pTa | no | Recurrence of a previuos NMI carcinoma. No follow-up and vital status information. | + | + |
| #4 | M | 63 | Non-invasive papillary urothelial carcinoma | low | pTa | no | Recurrence of 4 previuos NMI carcinomas. No follow-up and vital status information. | + | + |
| #5 | F | 75 | Non-invasive papillary urothelial carcinoma | low | pTa | no | n.a. | + | + |
| #6 | M | 66 | Non-invasive papillary urothelial carcinoma | low | pTa | no | n.a. | + | + |
| #7 | M | 83 | Non-invasive papillary urothelial carcinoma | low | pTa | no | n.a. | + | + |
| #8 | M | 67 | Non-invasive papillary urothelial carcinoma | low | pTa | no | Subsequent cystectomy and ureterocutaneostomy.  No vital status information. | + | + |
| #9 | M | 60 | Non-invasive papillary urothelial carcinoma | low | pTa | no | Two subsequent NMI relapses.  No vital status information. | + | + |
| #10 | M | 77 | Non-invasive papillary urothelial carcinoma | low | pTa | no | Recurrence of 2 previous NMI tumors;  3 subsequent relapses (2 NI, the last one pT4a pN0).  No vital status information. | + | + |
| #11 | F | 82 | Infiltrating urothelial carcinoma | high | pT4 | Muscle, vagina, vascular | Recurrence of a previous NMI carcinoma. No follow-up and vital status information. | + | + |
| #12 | M | 93 | Infiltrating urothelial carcinoma | high | n.a. | Muscle | One subsequent relapse (MI tumor).  No vital status information. | + | + |
| #13 | M | 85 | Infiltrating urothelial carcinoma | high | pT1 | Chorion | 2 subsequent relapses (NMI tumors).  No vital status information. | + | + |
| #14 | M | 75 | Infiltrating urothelial carcinoma | high | pT1 | Chorion | Simultaneous kidney tumor and lung metastasis. No vital status information. | + | + |
| #15 | M | 68 | Infiltrating urothelial carcinoma | high | pT1 | Chorion | Subsequent carcinoma in situ.  No vital status information. | + | + |
| #16 | M | 86 | Infiltrating urothelial carcinoma | high | pT1 | Chorion | Subsequent lung carcinoma.  No vital status information. | + | + |
| #17 | M | 73 | Infiltrating urothelial carcinoma | high | ypT2a | Muscle, vascular | Recurrence of 2 previous MI tumors. Subsequent cystectomy.  No vital status information. | + | n.a. |
| #18 | M | 67 | Infiltrating urothelial carcinoma | high | pT1 | Chorion | n.a. | + | n.a. |
| #19 | M | 76 | Infiltrating urothelial carcinoma | high | pT4 | Adipose tissue, seminal vesicle, vascular | Subsequent cystectomy.  No vital status information. | + | n.a. |

NMI: non-muscle invasive, MI: muscle-infiltrating, B: biopsy, CSC: cancer stem cells, na: not available

| SEX | AGE AT SURGERY | HISTOTYPE | GRADE | AJCC PATHOLOGIC T | AJCC PATHOLOGIC N | CLINICAL DATA |
| --- | --- | --- | --- | --- | --- | --- |
|  |  |  |  |  |  |  |
| Male: 73.8%  Female: 26.2% | 34.4 to < 45.5: 1.94%  45.5 to < 56.7: 11.41%  56.7 to < 67.8: 33.25%  67.8 to < 78.9: 35.19%  78.9 to < 90: 17.96% | Transitional cell carcinoma: 83.25%  Papillary transitional cell carcinoma: 16%  Others: 0.75% | n.a. | t0: 0.24%  t1: 0.73%  t2: 29.12%  t3: 47.57%  t4: 14.32%  tx: 0.24%  missing: 7.77% | n0: 58.01%  n1: 11.41%  n2: 18.45%  n3: 1.94%  nx: 8.74%  missing : 1.46% | Alive: 55.6%  Dead: 44.2%  n.a.: 0.2% |

**Table S1b**. TCGA cohort’s data.
