# Supplementary material for: Analysis of copy number alterations in bladder cancer stem cells revealed a prognostic role of LRP1B
Source: World J Urol. 2022 Jul 16;40(9):2267–73. doi: 10.1007/s00345-022-04093-1 (PMC9287687; doi:10.1007/s00345-022-04093-1)
Supplement: Supplementary file 4 — Supplementary file4 (DOCX 30 KB) [file 345_2022_4093_MOESM4_ESM.docx]

| GENES | COPY NUMBER GAIN FREQUENCY (%) | | | | | COPY NUMBER LOSS FREQUENCY (%) | | | | |
| --- | --- | --- | --- | --- | --- | --- | --- | --- | --- | --- |
|  | GDC DATA (n=408) | NMIBCs | | MIBCs | | GDC DATA (n=408) | NMIBCs | | MIBCs | |
|  |  | B (10) | C (10) | B (9) | C (6) |  | B (10) | C (10) | B (9) | C (6) |
| *AATF* | 7,1  (29) | 0 | 10  (1) | 11,1  (1) | 0 | 3,6  (15) | 0 | 0 | 0 | 0 |
| *AHR ^§^* | 9,8  (40) | 10  (1) | 10  (1) | **44,4***  (4) | 16,7  (1) | 1,5  (6) | 10  (1) | 0 | 0 | 0 |
| *ANXA7 ^§^* | 3,7  (15) | 0 | 0 | 0 | 0 | 3,9  (16) | 10  (1) | 10  (1) | 22,2  (2) | 0 |
| *ARID1A* | 2,9  (12) | 0 | 0 | 0 | 0 | 8,8  (36) | 10  (1) | 0 | 0 | 0 |
| *ATE1* | 3,4  (14) | 0 | 0 | 11,1  (1) | **33,3***  (2) | 5,9  (24) | 0 | 0 | 11,1  (1) | 0 |
| *BIRC3* | 7,6  (31) | 0 | 0 | 0 | 33,3  (2) | 3,7  (15) | 10  (1) | 20  (2) | 11,1  (1) | 16,7  (1) |
| *CDH1* | 9,5  (39) | 0 | 0 | 0 | 16,7  (1) | 1,9  (8) | 0 | 0 | 11,1  (1) | 0 |
| *CDKAL1* | 27,9  (114) | 10  (1) | 0 | 55,6  (5) | 33,3  (2) | 2,7  (11) | 10  (1) | **40***  (4) | 0 | 16,7  (1) |
| *CUL3* | 1,2  (5) | 10  (1) | 0 | 0 | 0 | 22,3  (91) | 10  (1) | 10  (1) | 22,2  (2) | 16,7  (1) |
| *DDB2* | 5,6  (23) | 0 | 0 | 0 | 0 | 3,2  (13) | 10  (1) | 0 | 0 | 16,7  (1) |
| *EPHA3* | 6,4  (26) | 0 | 0 | 11,1  (1) | 0 | 6,9  (28) | **30***  (3) | 10  (1) | 11,1  (1) | 0 |
| *FGF3* | 23,8  (97) | 30  (3) | **40***  (4) | 33,3  (3) | 16,7  (1) | 0,7  (3) | 0 | 10  (1) | 11,1  (1) | 0 |
| *FHIT* | 4,2  (17) | 0 | 0 | 11,1  (1) | 0 | 15,4  (63) | 30  (3) | 20  (2) | **44,4***  (4) | 50  (3) |
| *FHL2 ^§^* | 4,4  (18) | 20  (2) | 10  (1) | 0 | 0 | 2,7  (11) | 10  (1) | 0 | 11,1  (1) | 0 |
| *GATA3* | 18,1  (74) | 0 | 0 | 0 | 0 | 0,7  (3) | 0 | 0 | 0 | 0 |
| *HIPK3* | 3,9  (16) | 0 | 0 | 0 | 0 | 2,7  (11) | **20***  (2) | 0 | **22,2***  (2) | 0 |
| *IKZF2* | 5,6  (23) | 10  (1) | 0 | 0 | 0 | 20,6  (84) | 10  (1) | 0 | 44,4  (4) | 33,3  (2) |
| *IPO11* | 1,9  (8) | 10  (1) | 0 | 0 | 0 | 8,8  (36) | 0 | 10  (1) | **33,3***  (3) | 16,7  (1) |
| *KIAA0196* | 14,4  (59) | 0 | 0 | 0 | 0 | 2,4  (10) | 0 | 0 | 0 | 0 |
| *KRAS ^§^* | 5,9  (24) | 10  (1) | 10  (1) | 0 | **33,3***  (2) | 2,2  (9) | **20***  (2) | 10  (1) | 0 | 0 |
| *LRP1B* | 4,9  (20) | 0 | 0 | 0 | 0 | 18,1  (74) | 40  (4) | 10  (1) | **77,8***  (7) | **66,7***  (4) |
| *PABPC1* | 23,5  (96) | 0 | 0 | 11,1  (1) | 16,7  (1) | 0,5  (2) | 0 | 0 | 0 | 0 |
| *PDE4D* | 5,6  (23) | 10  (1) | 0 | 0 | 0 | 20,1  (82) | 20  (2) | 10  (1) | **55,6***  (5) | 50  (3) |
| *PRKCI* | 11  (45) | 0 | 0 | 11,1  (1) | 0 | 0,5  (2) | 0 | 0 | 0 | 0 |

| GENES | COPY NUMBER GAIN FREQUENCY (%) | | | | | COPY NUMBER LOSS FREQUENCY (%) | | | | |
| --- | --- | --- | --- | --- | --- | --- | --- | --- | --- | --- |
|  | GDC DATA (n=408) | NMIBCs | | MIBCs | | GDC DATA (n=408) | NMIBCs | | MIBCs | |
|  |  | B (10) | C (10) | B (9) | C (6) |  | B (10) | C (10) | B (9) | C (6) |
| *PTCH2 ^§^* | 5,4  (22) | 0 | 0 | 0 | 0 | 1,7  (7) | 10  (1) | **30***  (3) | 0 | 0 |
| *RAF1* | 20,6  (84) | 30  (3) | 10  (1) | 44,4  (4) | 33,3  (2) | 0,7  (3) | 0 | **10***  (1) | 0 | 0 |
| *SEMA3E* | 3,7  (15) | 10  (1) | 10  (1) | **33,3***  (3) | 16,7  (1) | 1,9  (8) | 10  (1) | 0 | 0 | 0 |
| *TGFB2* | 5,6  (23) | 0 | 10  (1) | 11,1  (1) | 0 | 5,9  (24) | 0 | 0 | 11,1  (1) | 0 |
| *TMPRSS2* | 4,2  (17) | 0 | 0 | 0 | 0 | 3,2  (13) | 10  (1) | **40***  (4) | 0 | 0 |
| *TOP2A* | 8,3  (34) | 0 | 0 | 22,2  (2) | 33,3  (2) | 2,2  (9) | 0 | 0 | 0 | 0 |
| *TSC1* | 4,9  (20) | 0 | 0 | 0 | 0 | 1,7  (7) | **40***  (4) | 10  (1) | 11,1  (1) | **33,3***  (2) |
| *TSHZ3* | 10,5  (43) | 10  (1) | 0 | 11,1  (1) | 33,3  (2) | 1,7  (7) | 0 | 10  (1) | 0 | **33,3***  (2) |
| *WWOX* | 9,1  (37) | 10  (1) | 10  (1) | 0 | 0 | 14,7  (60) | 20  (2) | 40  (4) | **77,8***  (7) | 16,7  (1) |
| *ZNF706* | 23,5  (96) | 0 | 10  (1) | 33,3  (3) | **66,7***  (4) | 0,5  (2) | 0 | 0 | 0 | 0 |

*^§^* 2 probes for the gene; B: biopsies; C: cancer stem cells; NMIBC: non-muscle invasive bladder cancer; MIBC: muscle infiltrating bladder cancer. Frequencies are expressed in percentage and the number of cases is indicated within brackets. * p<0,05 Chi-square or Fisher's exact test
