# Supplementary material for: Analysis of copy number alterations in bladder cancer stem cells revealed a prognostic role of LRP1B
Source: World J Urol. 2022 Jul 16;40(9):2267–73. doi: 10.1007/s00345-022-04093-1 (PMC9287687; doi:10.1007/s00345-022-04093-1)
Supplement: Supplementary file 5 — Supplementary file5 (DOCX 31 KB) [file 345_2022_4093_MOESM5_ESM.docx]

**Table S4**: Survival data of TCGA patients.

| **Gene** | Cases with CNAs n° | | | | **6 months survival** | | | | **1 year survival** | | | | **5 years survival** | | | | **Prior malignancy** | | | |  |
| --- | --- | --- | --- | --- | --- | --- | --- | --- | --- | --- | --- | --- | --- | --- | --- | --- | --- | --- | --- | --- | --- |
|  |  |  |  |  | **Gain** | | **Loss** | | **Gain** | | **Loss** | | **Gain** | | **Loss** | | **Gain** | | **Loss** | |  |
|  | Gain | Loss | | | Alive | Dead | Alive | Dead | Alive | Dead | Alive | Dead | Alive | Dead | Alive | Dead | No | Yes | No | Yes |  |
| ***AATF*** | 29 | 15 | | | 29/29  100% | 0/29  0% | 14/15  93,3% | 1/15  0,7% | 23/29  79,3% | 5/29  20,7% | 12/15  80% | 3/15  20% | 14/29  48,3% | 15/29  51,7% | 12/15  80% | 3/15  20% | 20/29  69% | 9/29  31% | 13/15  86,6% | 2/15  13,4% |  |
| ***AHR*** | 40 | 6 | | | 36/40  90% | 4/40  10% | 6/6  100% | 0/6  0% | 30/40  75% | 10/40  25% | 6/6  100% | 0/6  0% | 21/40  52,5% | 19/40  47,5% | **6/6**  **100%*** | **0/6**  **0%** | 25/40  62,5% | 15/40  37,5% | 4/6  66,7% | 2/6  33,3% |  |
| ***ANXA7*** | 15 | 16 | | | 13/15  86,6% | 2/15  13,4% | 14/16  87,5% | 2/16  12,5% | 12/15  80% | 3/15  20% | 13/16  81,2% | 3/16  18,8% | 7/15 46,7% | 8/15  53,3% | 10/16 62,5% | 6/16  37,5% | 10/15  66,7% | 5/15  33,3% | 13/16  81,2% | 3/16  18,8% |  |
| ***ARID1A*** | 12 | 36 | | | 11/12  91,7% | 1/12  8,3% | 33/36  91,7% | 3/36  8,3% | 10/12  83,3% | 2/12  16,7% | 27/36  75% | 9/36  25% | 8/12 66,7% | 4/12  33,3% | 16/36 44,4% | 20/36  55,6% | 6/12  50% | 6/12  50% | 30/36  83,3% | 6/36  16,7% |  |
| ***ATE1*** | 14 | 24 | | | 12/14  85,7% | 2/14  14,3% | 22/24  91,7% | 2/24  8,3% | 11/14  78,6% | 3/14  21,4% | 16/24  66,7% | 8/24  33,3% | 10/14 71,4% | 4/14  28,6% | 10/24  41,7% | 14/24  58,3% | 12/14  85,7% | 2/14  14,3% | 18/24  75% | 6/24  25% |  |
| ***BIRC3*** | 31 | 15 | | | 30/31  96,8% | 1/31  3,2% | 13/15  86,6% | 2/15  13,4% | 27/31  87,1% | 4/31  12,9% | 11/15  73,3% | 4/15  26,7% | **24/31**  **77,4%*** | **7/31**  **22,6%** | 8/15  53,3% | 7/15 46,7% | 26/31  83,9% | 5/31  16,1% | 12/15  80% | 3/15  20% |  |
| ***CDH1*** | 39 | 8 | | | 34/39  87,2% | 5/39  12,8% | 7/8  87,5% | 1/8  12,5% | 28/39  71,8% | 11/39  28,2% | 6/8  75% | 2/8  25% | 21/39 53,8% | 18/39  46,2% | 5/8 62,5% | 3/8  37,5% | 26/39  66,7% | 13/39  33,3% | 4/8  50% | 4/8  50% |  |
| ***CDKAL1*** | 114 | 11 | | | 107/114  93,9% | 7/114  6,1% | 9/11  81,8% | 2/11  18,2% | 96/114  84,2% | 18/114  15,8% | 8/11  72,7% | 3/11  27,3% | **79/114**  **69,3%*** | **35/114**  **30,7%** | 5/11  45,5% | 6/11  54,5% | 78/114  68,4% | 36/114  31,6% | 10/11  90,9% | 1/11  9,1% |  |
| ***CUL3*** | 5 | 91 | | | 4/5  80% | 1/5  20% | 84/91  92,3% | 7/91  7,7% | 4/5  80% | 1/5  20% | 76/91  83,5% | 15/91  16,5% | 4/5  80% | 1/5  20% | 58/91  63,7% | 33/91  36,3% | 4/5  80% | 1/5  20% | 64/91  70,3% | 27/91  29,7% |  |
| ***DDB2*** | 23 | 13 | | | 22/23  95,7% | 1/23  4,3% | 12/13  92,3% | 1/13  7,7% | 19/23  82,6% | 4/23  17,4% | 12/13  92,3% | 1/13  7,7% | 16/23  69,6% | 7/23  30,4% | 10/13  76,9% | 3/13  23,1% | 14/23  60,9% | 9/23  39,1% | 9/13  69,2% | 4/13  30,8% |  |
| ***EPHA3*** | 26 | 28 | | | 23/26  88,5% | 3/26  11,5% | 26/28  92,9% | 2/28  7,1% | 23/26  88,5% | 3/26  11,5% | 22/28  78,6% | 6/28  21,4% | 16/26  61,5% | 10/26  38,5% | 19/28  67,9% | 9/28  32,1% | 18/26  69,2% | 8/26  30,8% | 20/28  71,4% | 8/28  28,6% |  |
| ***FGF3*** | 97 | 3 | | | 89/97  91,8% | 8/97  8,2% | 3/3  100% | 0/3  0% | 78/97  80,4% | 19/97  19,6% | 2/3  66,7% | 1/3  33,3% | 58/97  59,8% | 39/97  40,2% | 2/3  66,7% | 1/3  33,3% | 76/97  78,3% | 22/97  22,7% | 3/3  100% | 0/3  0% |  |
| ***FHIT*** | 17 | 63 | | | 16/17  94,1% | 1/17  5,9% | 59/63  93,7% | 4/63  6,3% | 15/17  88,2% | 2/17  11,8% | 52/63  82,5% | 11/63  17,5% | 9/17  52,9% | 8/17  47,1% | 39/63  61,9% | 24/63  39,1% | 14/17  82,3% | 3/17  17,7% | 48/63  76,2% | 15/63  23,8% |  |
| ***FHL2*** | 18 | | 11 | | 16/18  88,9% | 2/18  11,1% | 11/11  100% | 0/11  0% | 13/18  72,2% | 5/18  27,8% | 9/11  81,8% | 2/11  18,2% | 12/18  66,7% | 6/18  33,3% | 6/11  54,5% | 5/11  45,5% | 11/18  61,1% | 7/18  38,9% | 8/11  72,7% | 3/11  27,3% | |
| ***GATA3*** | 74 | | 3 | | 68/74  91,9% | 6/74  8,1% | 3/3  100% | 0/3  0% | 60/74  81,1% | 14/74  18,9% | 1/3  33,3% | 2/3  66,7% | 47/74  63,5% | 27/74  36,5% | 0/3  0% | 3/3  100% | 49/74  66,2% | 25/74  33,8% | 3/3  100% | 0/3  0% | |
| ***HIPK3*** | 16 | | 11 | | 15/16  93,8% | 1/16  6,2% | 10/11  90,9% | 1/11  9,1% | 14/16  87,5% | 2/16  12,5% | 10/11  90,9% | 1/11  9,1% | 9/16  56,3% | 7/16  43,7% | 9/11  81,8% | 2/11  18,2% | 12/16  75% | 4/16  25% | 10/11  90,9% | 1/11  9,1% | |
| ***IKZF2*** | 23 | | 84 | | 22/23  95,7% | 1/23  4,3% | 77/84  91,7% | 7/84  8,3% | 17/23  73,9% | 6/23  26,1% | 68/84  81% | 16/84  19% | 14/23  60,9% | 9/23  39,1% | 54/84  64,3% | 30/84  35,7% | 21/23  91,3% | 2/23  8,7% | 61/84  72,6% | 23/84  27,4% | |
| ***IPO11*** | 8 | | 36 | | 7/8  87,5% | 1/8  12,5% | 34/36  94,4% | 2/36  5,6% | 7/8  87,5% | 1/8  12,5% | 30/36  83,3% | 6/36  16,7% | 5/8 62,5% | 3/8  37,5% | 23/36 63,9% | 13/36  36,1% | 5/8 62,5% | 3/8  37,5% | 27/36  75% | 9/36  25% | |
| ***KIAA0196*** | 59 | | 10 | | 54/59  91,5% | 5/59  8,5% | 10/10  100% | 0/10  0% | 48/59  81,4% | 11/59  18,6% | 9/10  90% | 1/10  10% | 38/59 64,4% | 21/59  35,6% | 8/10 80% | 2/10  20% | 40/59  67,8% | 19/59  32,2% | 7/10  70% | 3/10  30% | |
| ***KRAS*** | 24 | | 9 | | 21/24  87,5% | 3/24  12,5% | 8/9  88,9% | 1/9  11,1% | 20/24  83,3% | 4/24  16,7% | 8/9  88,9% | 1/9  11,1% | 15/24  62,5% | 9/24  37,5% | 7/9  77,8% | 2/9  22,2% | 21/24  87,5% | 3/24  12,5% | 9/9  100% | 0/0  0% | |
| ***LRP1B*** | 20 | | 74 | | 18/20  90% | 2/20  10% | 65/74  87,8% | 9/74  12,2% | 17/20  85% | 3/20  15% | **52/74**  **70,3%*** | **22/74**  **29,7%** | 15/20  75% | 5/20  25% | 41/74  55,4% | 33/74  44,6% | 16/20  80% | 4/20  20% | 55/74  74,3% | 19/74  25,7% | |
| ***PABPC1*** | 96 | | 2 | | 87/96  90,6% | 9/96  9,4% | 2/2  100% | 0/2  0% | 74/96  77,1% | 22/96  22,9% | 2/2  100% | 0/2  0% | 57/96  59,4% | 39/96  40,6% | 2/2  100% | 0/2  0% | 72/96  75% | 24/96  25% | 2/2  100% | 0/2  0% | |
| ***PDE4D*** | 23 | | 82 | | 19/23  82,6% | 4/23  17,4% | 74/82  90,2% | 8/82  9,8% | 18/23  78,3% | 5/23  21,7% | 68/82  82,9% | 14/82  17,1% | 14/23  60,9% | 9/23  39,1% | 50/82  61% | 32/82  39% | 15/23  65,2% | 8/23  34,8% | 66/82  80,5% | 16/82  19,5% | |
| ***PRKCI*** | 45 | | 2 | | 41/45  91,1% | 4/45  8,9% | 2/2  100% | 0/2  0% | 34/45  75,6% | 11/45  24,4% | 2/2  100% | 0/2  0% | 22/45 48,9% | 23/45  51,1% | 1/2 50% | 1/2 50% | 31/45  68,9% | 14/45  31,1% | 1/2 50% | 1/2 50% | |
| ***PTCH2*** | 22 | | 7 | | 21/22  95,5% | 1/22  4,5% | 6/7  85,7% | 1/7  14,3% | 19/22  86,4% | 3/22  13,6% | 6/7  85,7% | 1/7  14,3% | 13/22 59,1% | 9/22  40,9% | 5/7 71,4% | 2/7  28,6% | 16/22  72,7% | 6/22  27,3% | 6/7  85,7% | 1/7  14,3% | |
| ***RAF1*** | 84 | | | 3 | 78/84  92,9% | 6/84  7,1% | 2/3  66,7% | 1/3  33,3% | 69/84  82,1% | 15/84  17,9% | 2/3  66,7% | 1/3  33,3% | 51/84 60,7% | 33/84  39,3% | 2/3 66,7% | 1/3  33,3% | 54/84  64,3% | 30/84  35,7% | 3/3  100% | 0/3  0% | |
| ***SEMA3E*** | 15 | | | 8 | 13/15  86,7% | 2/15  13,3% | 8/8  100% | 0/8  0% | 12/15  80% | 3/15  20% | 7/8  87,5% | 1/8  12,5% | 11/15 73,3% | 4/15  26,7% | 6/8 75% | 2/8  25% | 10/15  66,7% | 5/15  33,3% | 6/8 75% | 2/8  25% | |
| ***TGFB2*** | 23 | | | 24 | 20/23  87% | 3/23  13% | 20/24  83,3% | 4/24  16,7% | 17/23  73,9% | 6/23  26,1% | 19/24  79,2% | 5/24  20,8% | 12/23 52,2% | 11/23  47,8% | 15/24 62,5% | 9/24  37,5% | **12/23**  **52,2%** | **11/23**  **47,8%*** | 18/24  75% | 6/24  25% | |
| ***TMPRSS2*** | 17 | | | 13 | 15/17  88,2% | 2/17  11,8% | 12/13  92,3% | 1/13  7,7% | 15/17  88,2% | 2/17  11,8% | 12/13  92,3% | 1/13  7,7% | 10/17 58,8% | 7/17  41,2% | 8/13 61,5% | 5/13  38,5% | **7/17**  **41,2%** | **10/17 58,8%*** | **6/13**  **46,2%** | **7/13**  **53,8%*** | |
| ***TOP2A*** | 34 | | | 9 | 32/34  94,1% | 2/34  5,9% | 9/9  100% | 0/9  0% | 27/34  79,4% | 7/34  20,6% | 8/9  88,9% | 1/9  11,1% | 17/34 50% | 17/34  50% | 7/9 77,8% | 2/9  22,2% | 25/34  73,5% | 9/34  26,5% | 7/9 77,8% | 2/9  22,2% | |
| ***TSC1*** | 20 | | | 7 | 19/20  95% | 1/20  5% | 6/7  85,7% | 1/7  14,3% | 17/20  85% | 3/20  15% | 5/7 71,4% | 2/7  28,6% | 14/20 70% | 6/20  30% | 4/7 57,1% | 3/7  42,9% | 13/20  65% | 7/20  35% | 7/7  100% | 0/7  0% | |
| ***TSHZ3*** | 43 | | | 7 | 40/43  93% | 3/43  7% | 7/7  100% | 0/7  0% | 37/43  86% | 6/43  14% | 7/7  100% | 0/7  0% | 24/43 55,8% | 19/43  44,2% | 6/7 85,7% | 1/7  14,3% | 28/43  65,1% | 15/43  34,9% | 7/7  100% | 0/7  0% | |
| ***WWOX*** | 37 | | | 60 | 33/37  89,2% | 4/37  10,8% | **50/60**  **83,3%*** | **10/60**  **16,7%** | 29/37  78,4% | 8/37  21,6% | 44/60  73,3% | 16/60  26,7% | 21/37 56,8% | 16/37  43,2% | 34/60  56,7% | 26/60  43,3% | 25/37  67,6% | 12/37  32,4% | 49/60  81,7% | 11/60  18,3% | |
| ***ZNF706*** | 96 | | | 2 | 86/96  89,6% | 10/96  10,4% | 2/2  100% | 0/2  0% | 74/96  77,1% | 22/96  22,9% | 2/2  100% | 0/2  0% | 57/96 59,4% | 39/96  40,6% | 2/2 100% | 0/2  0% | 71/96  74% | 25/96  26% | 2/2  100% | 0/2  0% | |
| **GDC all cases (410)** | | | | | 6 months OS:  376 alive (91,7%), 33 dead (8,1%),  1 not reported (0,2%) | | | | 1 year OS: 330 alive (80,5%),  79 dead (19,3%),  1 not reported (0,2%) | | | | 5 years OS: 236 alive (57,6%),  173 dead (42,2%),  1 not reported (0,2%) | | | | Prior malignancy:  301 no (73,4%), 109 yes (26,6%) | | | | |

OS: overall survival; grey box: statistically significant difference between CN carriers and all cases
